# Predicting the Risk of Postoperative Complications in Patients Undergoing Minimally Invasive Resection of Primary Liver Tumors

**DOI:** 10.3390/jcm10040685

**Published:** 2021-02-10

**Authors:** Philipp K. Haber, Christoph Maier, Anika Kästner, Linda Feldbrügge, Santiago Andres Ortiz Galindo, Dominik Geisel, Uli Fehrenbach, Matthias Biebl, Felix Krenzien, Christian Benzing, Wenzel Schöning, Johann Pratschke, Moritz Schmelzle

**Affiliations:** 1 Department of Surgery, Campus Charité Mitte and Campus Virchow-Klinikum, Charité—Universitätsmedizin Berlin, Augustenburger Platz 1, 13353 Berlin, Germany; philipp.haber@charite.de (P.K.H.); anika.kaestner@charite.de (A.K.); linda.feldbruegge@charite.de (L.F.); santiago.ortiz-galindo@charite.de (S.A.O.G.); matthias.biebl@charite.de (M.B.); felix.krenzien@charite.de (F.K.); christian.benzing@charite.de (C.B.); wenzel.schoening@charite.de (W.S.); johann.pratschke@charite.de (J.P.); 2 Department of Radiology, Charité—Universitätsmedizin Berlin, Augustenburger Platz 1, 13353 Berlin, Germany; christoph.maier@charite.de (C.M.); dominik.geisel@charite.de (D.G.); uli.fehrenbach@charite.de (U.F.)

**Keywords:** laparoscopic liver surgery, hepatocellular carcinoma, cholangiocarcinoma, risk score

## Abstract

Minimal-invasive techniques are increasingly applied in clinical practice and have contributed towards improving postoperative outcomes. While comparing favorably with open surgery in terms of safety, the occurrence of severe complications remains a grave concern. To date, no objective predictive system has been established to guide clinicians in estimating complication risks as the relative contribution of general patient health, liver function and surgical parameters remain unclear. Here, we perform a single-center analysis of all consecutive patients undergoing laparoscopic liver resection for primary hepatic malignancies since 2010. Among the 210 patients identified, 32 developed major complications. Several independent predictors were identified through a multivariate analysis, defining a preoperative model: diabetes, history of previous hepatectomy, surgical approach, alanine aminotransferase levels and lesion entity. The addition of operative time and whether conversion was required significantly improved predictions and were thus incorporated into the postoperative model. Both models were able to identify patients with major complications with acceptable performance (area under the receiver-operating characteristic curve (AUC) for a preoperative model = 0.77 vs. postoperative model = 0.80). Internal validation was performed and confirmed the discriminatory ability of the models. An easily accessible online tool was deployed in order to estimate probabilities of severe complication without the need for manual calculation.

## 1. Introduction

Liver resection is the primary option for the curative treatment of hepatic malignancies [1,2,3]. While in some disease entities local ablative techniques such as radiofrequency ablation (RFA) and microwave ablation (MWA) can be effectively deployed to treat small tumors, they rapidly lose efficacy with increasing nodule size [4,5]. Until recently, liver surgery has been thought of as a classical domain of open surgery with the need for large incisions and inherent protracted postoperative courses. This is particularly evident in the case of malignancies, where concerns regarding oncologic outcomes have dampened the widespread application of minimal-invasive techniques. These concerns have been disproven, and laparoscopic liver resection (LLR) is, in select centers, applied as the mainstay treatment option independent of tumor size, entity or location [6,7,8]. Indeed, LLR has been shown to be an effective method to treat the most common primary hepatic malignancies, hepatocellular carcinoma (HCC) [9] and intrahepatic cholangiocarcinoma (iCC) [10,11].

Incremental improvements in technology and instruments as well as increasing experience in tailored surgical training [12] have contributed to LLR being regarded as mature for several distinct resection types [1,13]. The overarching theme of studies on LLR is that the minimal-invasive approach is associated with less intraoperative bleeding [14] and overall fewer complications [15,16,17] when compared to open resection, which is still burdened by potentially life-threatening complications [9,18]. Few reports, however, have identified risk factors for postoperative complications among patients undergoing LLR. Instead, emphasis has been placed on defining risk factors for conversion and subsequent prolonged courses [19,20]. Validated scoring systems appraising the expected difficulty of a resection type [21,22,23,24] are used in clinical practice, but available predictive systems that have been developed for the occurrence of complications have key shortcomings. First, some scores were developed using subjective interpretation by individual surgeons and have not been formulated in an unsupervised analysis [21]. Other groups focused on the type of resection without giving patient-dependent factors enough consideration [23]. Similarly, a recent comprehensive report generating and validating a model using objective criteria expanded the scope by considering all types of indications but did not factor in the general health of the patient. Thus, in these reports, the premise of the occurrence of complications shifts from being a complex interplay of patient history, surgical technique and experience to being a plain readout of the operative procedure.

The present study aimed to address this shortcoming by reporting risk factors for major complications in patients undergoing hepatectomy for primary malignancies. We herein define a logistic regression model factoring in solely preoperative variables as well as one that also considers intraoperative variables. Models are validated using a bootstrapping approach. Finally, in order to address the intrinsic shortcoming of logistic models, a lack of clinical utility due to the need for complex scoring, we deployed an online application providing probabilities for the occurrence of major complications.

## 2. Materials and Methods

Clinical courses of all consecutive patients undergoing LLR between January 2010 and May 2020 at the Department of Surgery, Campus Charité Mitte and Campus Virchow-Klinikum, Charité–Universitätsmedizin Berlin, Germany were analyzed for this retrospective study. Patients who had undergone resection for lesions other than primary liver malignancies were excluded from the analysis. The study was approved by the local Ethics Committee (EA2/006/16).

### 2.1. Patient Evaluation and Surgical Approaches

In all patients, preoperative workup included imaging with either triphasic contrast-enhanced computed tomography or magnetic resonance imaging as well as platelet count, liver and kidney function tests and, in the case of cirrhosis or planned major hepatectomy, maximum liver function capacity (LiMAx) testing. Indication for resection was given through a multidisciplinary hepatobiliary tumor board consisting of surgeons, hepatologists, medical oncologists and radiologists. Laparoscopic surgery was performed in the French position. Three different techniques were applied as described elsewhere [25,26,27]: standard multiport laparoscopy (MILL), single-incision laparoscopic surgery (SILS) or hand-assisted laparoscopic surgery (HALS). All resections were performed with curative intent.

### 2.2. Data Collection and Study Endpoints

Pre- and intraoperative variables were collected in a prospective database. For the purpose of the study, imaging from included patients was evaluated for signs indicative of liver cirrhosis and portal hypertension.

Complications were defined by the Clavien–Dindo classification system (CD) [28]. The primary endpoint was the occurrence of major postoperative complications defined as grade 3 and above. Mortality was assessed as a secondary endpoint. As the CD system defines the given grade as the most severe complication that occurred, patients that decease during hospitalization by default also have complications of grades 3 or 4. We therefore included these patients for the primary endpoint and analyzed them separately for the secondary endpoint. In accordance with the original classification system, morbidity and mortality were defined as complications and death occurring within 90 days after the procedure. Analysis was based on the intention-to-treat principle and, therefore, any procedure planned laparoscopically was included. Conversion was defined as switching from any laparoscopic technique to laparotomy.

### 2.3. Statistical Analysis

Analysis was performed using SPSS V22.0^®^ (IBM, Armonk, NY, USA) and R software, version 4.02. The distribution of continuous variables was evaluated with the Shapiro–Wilk test. A comparison of continuous variables in the case of nonparametric distribution was performed with the Mann–Whitney test. Continuous variables with a Gaussian distribution were compared with ANOVA. Categorical variables were compared using the Chi-square or Fisher exact test. A two-tailed value of *p* < 0.05 was considered statistically significant. Continuous variables are stated as the mean with the 95% confidence interval, whereas categorical variables are reported as counts with percentages in brackets unless stated otherwise.

Missing variables ranged from 0% up to 41% for AFP. A granular view of missing variables is shown in Figure S1. Multiple imputations using a regression-switching approach was applied in order to avoid omitting cases from the analysis (R package *mice*, with m = 20). Imputations were performed under the missing-at-random assumption for missing variables, using the ppm method for continuous variables and logreg for categorical variables. Imputed datasets were combined to generate estimates using Rubin’s rules. The stability of imputed variables was ensured through density plotting.

The model was constructed using intraoperative and preoperative variables and testing the association with the above-defined complication categories along with a bivariate logistic regression model with odds ratios (ORs) to capture differences between cases with major complications from those without. The integrated continuous variable had a linear relationship with the outcome and was applied as a linear term. Categorical and continuous variables associated with the occurrence of major complications in a univariate model (*p* < 0.1) were included in a multivariate logistic regression model with proportional OR using a removal significance of *p* = 0.05. We constructed a preoperative and postoperative model including respective variables.

Ultimately, the performance of the two models was evaluated by ascertaining the area under the ROC curve (AUC) to discriminate major complications (CD 3-5) from no or minor complications (CD 0-2). Pre- and postoperative models were compared using the bootstrap test. We defined a threshold of an AUC greater than 0.6 to capture acceptable performance. The goodness of fit of the model was tested using the Hosmer–Lemeshow test. Finally, internal validation of the models was performed by bootstrapping 3000 datasets.

## 3. Results

### 3.1. Cohort Characteristics

Screening of patients undergoing LLR between January 2010 and May 2020 identified 210 out of 572 patients with a primary hepatic malignancy as an indication. Expectedly, the majority of resections were performed for HCC (73.8%) and iCC (20.0%). Most cases presented without viral hepatitis as a cause of the underlying liver disease (63.8%). Liver function was well compensated in the included patients, as accounted for by Child–Pugh scores up to B7 in all cases and a median MELD score of 8 (95% CI = 7.81–8.50). Preoperative imaging was suspicious for liver cirrhosis in 69.5% of cases, whereas radiographic signs of portal hypertension were observed in 29.9%. Major hepatectomy was performed in 24.8% of all patients, and MILL was the most frequently applied surgical approach (76.6%). Baseline patient characteristics as well as intraoperative data are presented based on the occurrence of major complications (CD 3-5) in Table 1. As no data were available on blood loss, we used the number of perioperatively transfused red blood cell (RBC) concentrates as a surrogate. Overall morbidity comprising all CD stages was 31.4%, whereas 32 patients (15.2%) had major complications. Four patients (1.9%) succumbed to postoperative complications. The most common indication for reoperation was a burst abdomen, which occurred in three patients (two HALS cases, one MILL case). Major complications are detailed in Table 2.

### 3.2. Model Generation for the Prediction of Major Complications

Out of the 10 preoperative variables associated with major complications in the univariate analysis, 5 variables were retained for the preoperative risk model after the multivariate analysis (Table 3): preoperative alanine aminotransferase (ALT) levels, history of previous liver resection (yes/no), diabetes (yes/no), resection performed as MILL (yes/no) and whether or not the malignancy was an HCC (yes/no). Major complications were associated with high ALT levels, previous hepatectomies, diabetes, application of techniques other than MILL and non-HCC lesions.

For the postoperative setting, duration of surgery and the need for conversion to laparotomy, both associated with an adverse safety outcome, were included in the model as well. The entity of the diagnosis was dropped from the model as it did not retain statistical significance. Model calibration parameters are shown in Figure S2 and the regression coefficients per variable in Table S1.

### 3.3. Prediction of Pre- and Postoperative Model for Primary and Secondary Endpoints

**Figure 1:**
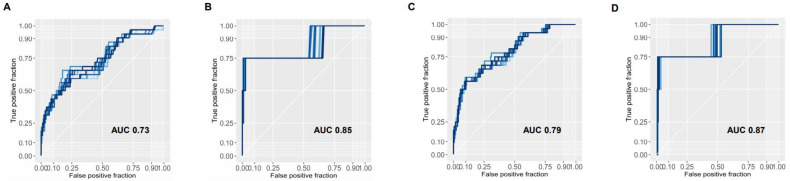
Performance of preoperative and postoperative models across imputed datasets Areas under the curves are shown for the preoperative model as well as the postoperative model for the primary endpoint of occurrence of major complications (**A**,**C**) and the secondary endpoint of mortality (**B**,**D**). Every curve represents one iteration of 20 imputed datasets for missing variables. The mean AUC for each model is depicted.

The preoperative model was capable of discriminating CD 3-5 from CD 0-2 patients with a mean AUC across imputations of 0.73 (Figure 1A). Applying the same model to the endpoint of mortality, it retained its predictive ability at an AUC of 0.85 (Figure 1B). Likewise, the AUC for the postoperative model to identify major complications was 0.79 (Figure 1C) and 0.87 when considering mortality as an endpoint (Figure 1D). Bootstrap testing revealed the postoperative model to be significantly more accurate for both the primary and secondary endpoints (*p* < 0.0001 and *p* < 0.01, for major complications and mortality, respectively).

To address the evident risk of overfitting the model to the data, we performed internal validation by bootstrapping (random sampling with replacement) 3000 datasets with replacement. After correction for overoptimism, the AUC for the preoperative model was 0.72 and 0.76 for the postoperative model. The Hosmer–Lemeshow test revealed an acceptable goodness of fit for the preoperative model (*p* = 0.22) and the postoperative model (*p* = 0.59).

### 3.4. Predictive Ability of the Two Models in Patients Undergoing Major Hepatectomy

Fifty-two patients (24.8%) underwent major hepatectomy (33 right (extended) hemihepatectomies, 19 left (extended) hemihepatectomies). Applying the models created in the entire cohort to these patients, the AUC for predicting major complications of the preoperative model was 0.67, whereas the AUC for the postoperative model was 0.77. The bootstrap test revealed, for the entire dataset, the postoperative model to be significantly more accurate in predicting morbidity. When considering the secondary endpoint of mortality, the AUC of both the preoperative and the postoperative model was 0.75.

### 3.5. Comparison of Predictive Models with Previously Reported Model

**Figure 2:**
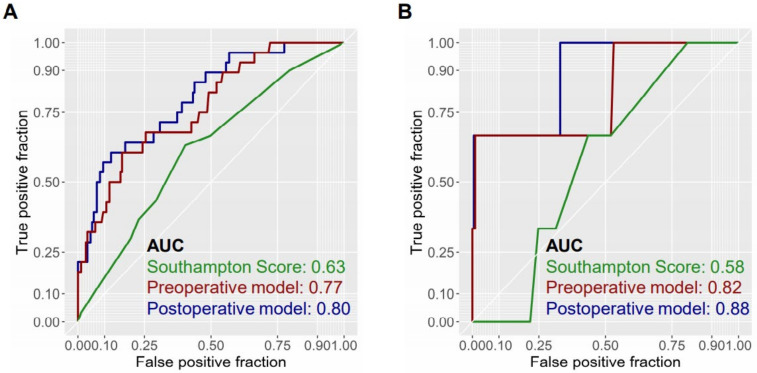
Comparison of predictive models. Comparative analysis of the AUC of the preoperative (red) and postoperative (blue) models compared to the Southampton score (green) both for the primary endpoint of major complications (**A**) and the secondary endpoint of mortality (**B**) reveals the two defined models to have a higher predictive value. AUC computation and model generation are based on the dataset, omitting cases with missing variables.

The discriminatory ability of both the preoperative and the postoperative model prompted us to compare the predictive ability with the Southampton Laparoscopic Liver Difficulty Score, a recently published scoring system from a multicenter cohort that, of note, predicts intraoperative complications [24]. The application of this system yielded an AUC of 0.63 as compared to the median AUC of 0.77 from our preoperative model and 0.80 of our postoperative model, in which nonimputed data are used for model generation (Figure 2). In accordance with this, the superior accuracy of the present models was maintained when mortality was used as the endpoint (AUC = 0.58 vs. 0.82 vs. 0.88 for the Southampton score, preoperative model and postoperative model, respectively).

## 4. Discussion

The rise of minimal-invasive techniques in hepatobiliary surgery is in full swing. More and more centers are adopting various approaches in their clinical practice, no longer being constrained by reservations regarding intraoperative safety and oncological outcome. With increasing experience, our understanding of the benefits of minimal-invasive techniques has evolved, and it is commonly regarded as settled knowledge that LLR is able to elicit faster functional recovery, reducing hospital stay and lowering morbidity rates while achieving noninferior outcomes from an oncological perspective. The application of LLR has opened up curative resections to patients who previously might have been considered inoperable due to impaired liver function or poor general health. In a very recent study, laparoscopy has been shown in a French multicenter cohort to reduce the risk of posthepatectomy liver failure [29]. The spectrum of potential complications after liver surgery, of course, goes beyond hepatic dysfunction. Indeed, preoperative patient health and liver function are critical, but likewise, the amount of tissue resected, type of resection and tumor entity are critical factors. Moreover, intraoperative decision making impacts outcome, and some complications may be attributable to low experience. In this regard, a structured curriculum to obtain necessary experience, as recently suggested, is vital [12]. With growing surgeon experience, however, other factors drive the occurrence of complications.

In the present study, we have examined resections of primary hepatic malignancies and developed two simple robust scoring systems, capable of predicting the occurrence of major complications. While similar approaches have been undertaken by other studies [21,24], they are limited by either biased model development or by placing disproportionate focus on technical factors rather than the patient or are only predicting intraoperative complications. The tools developed in the present report integrate parameters of general patient health, liver function and operative course that have been shown to be independently associated with an adverse outcome. Inclusion of operative time and the need for conversion, information immediately obtainable after surgery, improved the predictive ability substantially. Specifically, operative time is a powerful readout of aggressiveness of the surgical approach and highly predictive of the occurrence of postoperative complications. Likewise, conversion can be regarded as a surrogate of procedure complexity. By defining patients at risk of developing potentially life-threatening events after surgery, the models may help to improve patient selection for resection and identify patients requiring close monitoring in the postoperative period. Moreover, the model can be applied in the process of training, as patients at high risk, where margins of error decrease, could be operated by fellows at later training stages.

Interestingly, no statistically meaningful correlation was observed between the presence of cirrhosis and the occurrence of major complications. This was the case both for preoperative imaging-based assessment as well as postoperative pathology. Moreover, factors associated with cirrhosis such as higher serum bilirubin, lower albumin and low thrombocyte counts showed no significant correlation with CD 3-5 complications. This may be, at least in part, attributable to the limited spectrum of hepatic dysfunction in included patients and the low likelihood of major resection carried out in these at-risk cases [30]. Except for one patient with a Child–Pugh B7 score, all patients were within Child–Pugh A. It should be considered, however, that the decreased magnitude of harm inflicted by LLR clouds the relationship between hepatic dysfunction and occurrence of complications in this patient subset and a trend might be more evident in (a) patients undergoing laparotomy or (b) patients with less compensated hepatic function, i.e., Child–Pugh B7 patients that undergo major hepatectomy.

The use of logistic regression models in clinical practice is constrained by the need to fill out complex formulas. Any models are therefore regarded with caution due to limited practicality. To address this, we have deployed our tool as an easily accessible online application providing an instant estimation of risk based on the provided input [31].

The limitations of this study are certainly its retrospective design and the single-center nature of the report. Moreover, as validation is only performed internally, the tools are still to a certain extent subjected to overfitting and thus external prospective validation is required. The lack of perfect prediction by the tools also shows that other factors, unaccounted for by either model, impact the outcome.

## 5. Conclusions

Predicting major morbidity has implications for surgical training, selecting the appropriate operative approach and postoperative clinical management. In this large-scale single-center study, we defined a robust logistic regression model, capable of predicting major postoperative complications, that incorporates individual data on lesion characteristics, patients’ general health, liver function and the surgical approach. The addition of intraoperative information significantly improves the predictive ability. This system outperforms previously reported scores that have failed to account for risk factors on the side of the patient as well as the operative procedure.

## Figures and Tables

**Table 1:** Patient characteristics based on the occurrence of major complications.

	Complication Grading According to Clavien–Dindo			
	CD 0-2 (*n* = 178)	CD 3-5 (*n* = 32)	Odds Ratio (95% CI)	*p*
General Variables				
Age in years	67.1 (65.6–68.7)	65.9 (61.6–70.1)	0.981 (0.949–1.016)	0.26
Male gender	121 (68.0)	22 (68.8)	1.036 (0.471–2.421)	0.93
BMI in kg/m^2^	26.8 (26.1–27.5)	26.5 (24.7–28.4)	0.999 (0.92–1.082)	0.98
Diabetes	49 (27.7)	14 (43.8)	2.047 (0.934–4.425)	0.07
HCC	136 (76.4)	19 (59.4)	0.451 (0.207–1.0)	0.047
HCV	37 (21.3)	11 (36.7)	2.144 (0.914–4.847)	0.07
HBV	23 (13.2)	3 (10.0)	0.729 (0.165–2.288)	0.63
Previous hepatectomy	8 (4.6)	4 (13.3)	3.935 (1.119–12.712)	0.02
Previous abdominal surgery	84 (47.2)	17 (53.1)	1.268 (0.596–2.723)	0.54
ASA 3/4	97 (55.4)	22 (68.8)	1.769 (0.791–3.956)	0.16
Surgical variables				
Number of previously performed LLRs	279 (255–302)	268 (210–326)	1.0 (0.997–1.002)	0.72
Major hepatectomy	40 (22.7)	12 (37.5)	2.04 (0.899–4.489)	0.08
Simultaneous ablation	5 (2.8)	3 (9.4)	3.579 (0.704–15.402)	0.09
MILL	143 (80.3)	17 (54.8)	0.315 (0.143–0.701)	0.004
HALS	31 (17.4)	12 (38.7)	1.362 (0.535–3.183)	0.49
SILS	4 (2.2)	2 (6.5)	2.9 (0.390–15.552)	0.23
Length of surgery (LOS) in min.	226.9 (213.6–240.1)	296.7 (260.4–333.0)	1.008 (1.004–1.012)	<0.001
Conversion	1 (0.6)	2 (6.3)	11.8 (1.097–258.613)	0.046
R1 Status	15 (8.6)	10 (31.3)	4.818 (1.891–12.011)	<0.001
Perioperative RBCs transfused	0.11 (0.0–0.21)	0.31 (0.0–0.65)	3.821 (0.736–16.915)	0.18
Maximum tumor diameter in cm	4.5 (4.0–4.9)	4.2 (3.4–5.1)	0.974 (0.848–1.10)	0.69
Liver function variables				
ALT, U/L	41.3 (33.8–48.9)	52.1 (26.8–77.3)	1.008 (1.0–1.016)	0.037
AST, U/L	48.4 (38.9–58.0)	56.7 (22.7–90.7)	1.004 (0.997–1.01)	0.25
Thrombocytes	192.1 (173.8–210.5)	200.5 (139.9–261.22)	1 (0.995–1.004)	0.98
Thrombocytes <100/uL	18 (10.3)	7 (23.3)	2.638 (0.941–6.809)	0.052
Albumin mg/dl	41.2 (40.0–42.4)	40.0 (38.4–42.7)	0.979 (0.901–1.070)	0.62
Bilirubin mg/dl	0.67 (0.59–0.76)	0.63 (0.51–0.75)	0.772 (0.249–1.59)	0.59
INR	1.1 (1.08–1.13)	1.1 (1.07–1.16)	3.772 (0.098–107.931)	0.45
ALBI score	−2.85 (−2.97−(−2.74))	−2.76 (−3.06–(−2.48))	1.278 (0.516–2.935)	0.575
FIB−4	3.24 (2.83–3.66)	3.28 (2.25–4.3)	1.005 (0.853–1.145)	0.95
LiMAX µg/kg/h	322 (289–355)	379 (306–451)	1.002 (0.999–1.005)	0.15
Cirrhosis in imaging	119 (70.8)	18 (62.1)	0.674 (0.30–1.570)	0.35
Cirrhosis in pathology	98 (58.0)	14 (46.7)	0.634 (0.287–1.385)	0.25
Advanced fibrosis (grade III-IV)	115 (68.0)	20 (66.7)	0.939 (0.412–2.143)	0.88
Portal Hypertension in imaging	50 (29.8)	9 (31.0)	1.062 (0.434–2.435)	0.89
MELD	8.2 (7.7–8.6)	9.1 (6.9–11.3)	1.077 (0.922–1.234)	0.30
Preoperative ascites	2 (1.3)	1 (3.8)	3.14 (0.142–33.970)	0.36

Variables are expressed as the mean with the 95% CI for continuous variables and as counts with percentages in brackets for categorical variables. An overview of missing data is shown in Figure S1. For continuous variables, the odds ratios are per one unit increase.

**Table 2:** Description of major complications.

Clavien–Dindo Grade	Frequency
3a	17 (8.1%)
Biliary leakage	6
Intraabdominal abscess	5
Pleural effusion	3
Pneumothorax	2
Wound infection	1
3b	11 (5.2%)
Biliary leakage	2
Burst abdomen	3
Ileus	1
Intraabdominal abscess	2
Postoperative hemorrhage	1
Wound infection	2
4	0 (0%)
5	4 (1.9%)
Congestive heart failure	1
Pneumonia	1
Pulmonary embolism	1
ISGLS C post hepatectomy liver failure	1

**Table 3:** Variables retained after the multivariate analysis.

	Odds Ratio	*p* Value
Preoperative model		
Diabetes	2.74 (1.13–6.63)	0.026
Rehepatectomy	5.79 (1.46–22.92)	0.013
ALT	1.01 (1.0–1.02)	0.033
Non-HCC	3.27 (1.28–8.36)	0.014
Standard multiport approach	0.26 (0.11–0.63)	0.003
Postoperative variables		
Length of surgery (LOS)	1.01 (1.0–1.01)	0.002
Conversion	23.4 (1.57–350.1)	0.023

## Data Availability

The data presented in this study are available on request from the corresponding author.

## Supplementary Materials

The following are available online at https://www.mdpi.com/2077-0383/10/4/685/s1, Figure S1. Missing data. Figure S2. Model calibration. Table S1. Model coefficients are depicted for the preoperative and postoperative model.
