# Supplementary material for: Predicting the Risk of Postoperative Complications in Patients Undergoing Minimally Invasive Resection of Primary Liver Tumors
Source: J Clin Med. 2021 Feb 10;10(4):685. doi: 10.3390/jcm10040685 (PMC7916554; doi:10.3390/jcm10040685)
Supplement: Supplementary file 1 [file jcm-10-00685-s001.pdf]

Table S1. Model coefficients are depicted for the preoperative and postoperative model.

Variable	Coefficient
Preoperative Model	
Intercept	-2.20204
Diabetes	1.00214
Repeat hepatectomy	1.71543
ALT	0.01014
Non-HCC	1.10619
MILL approach	-1.32158
Postoperative model	
Intercept	-3.95018
Diabetes	0.84134
Repeat hepatectomy	1.93562
ALT	0.0092
MILL approach	-0.99688
Length of surgery	0.00725
Conversion performed	3.19237

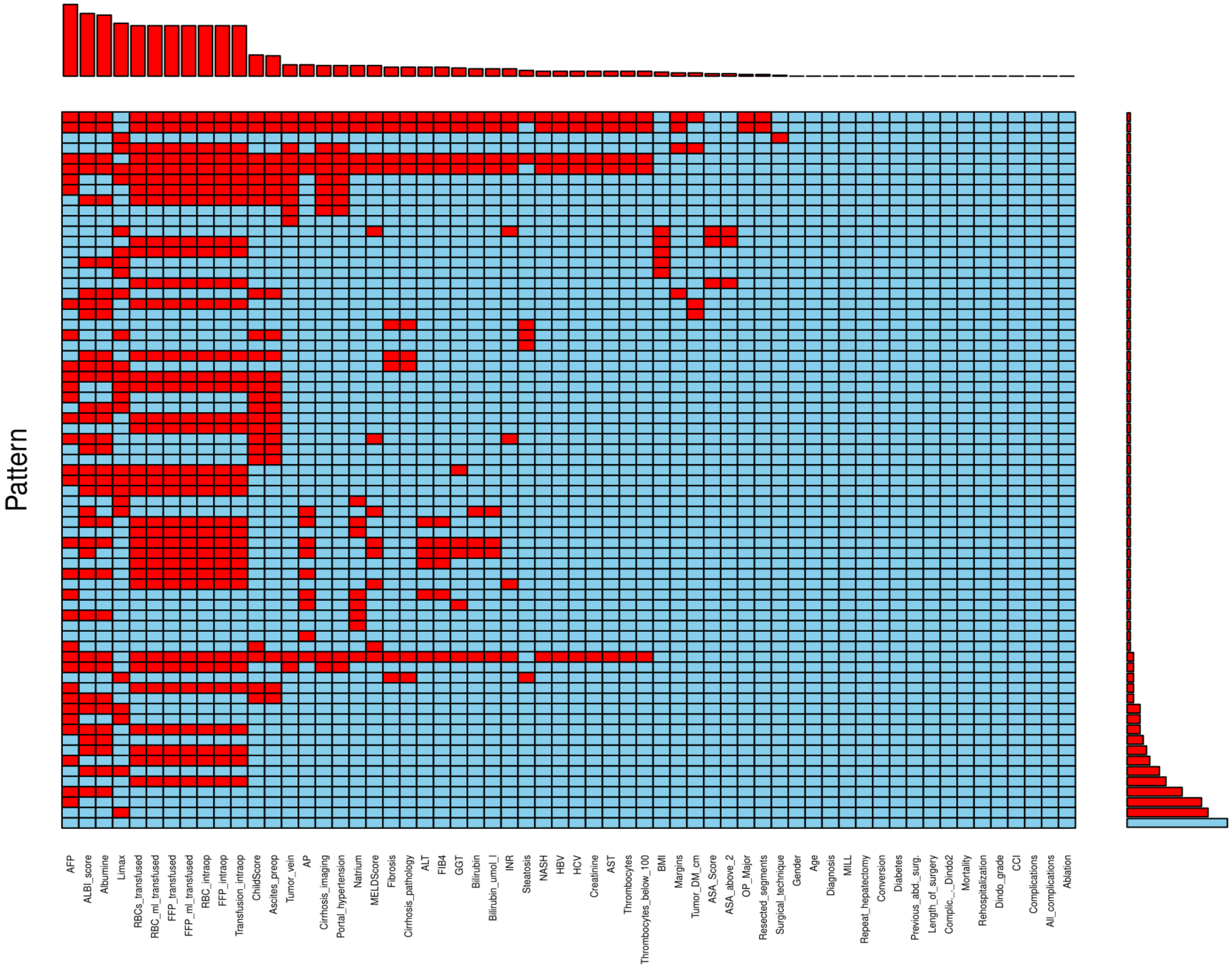
Pattern
AFP
ALBI_score
Albumine
Limax
RBCs_transfused
RBC_ml_transfused
FFP_transfused
FFP_ml_transfused
RBC_intraop
FFP_intraop
Transfusion_intraop
ChildScore
Ascites_preop
Tumor_vein
AP
Cirrhosis_imaging
Portal_hypertension
Natrium
MELDScore
Fibrosis
Cirrhosis_pathology
ALT
FIB4
GGT
Bilirubin
Bilirubin_umol_l
INR
Steatosis
NASH
HBV
HCV
Creatinine
AST
Thrombocytes
Thrombocytes_below_100
BMI
Margins
Tumor_DM_cm
ASA_Score
ASA_above_2
OP_Major
Resected_segments
Surgical_technique
Gender
Age
Diagnosis
MILL
Repeat_hepatectomy
Conversion
Diabetes
Previous_abd._surg.
Length_of_surgery
Complic._Dindo2
Mortality
Rehospitalization
Dindo_grade
CCI
Complications
All_complications
Ablation

Figure S1. Missing data. Patterns of missing data are depicted in red, available data in light blue. Columns represent individual variables, whereas rows represent distinct patterns of missing information. Frequency of the observed pattern is depicted in each row. Most frequently missing data where AFP and ALBI score. Imputation of missing data was performed using the R mice package with 20 imputed datasets that were used to generate the models.

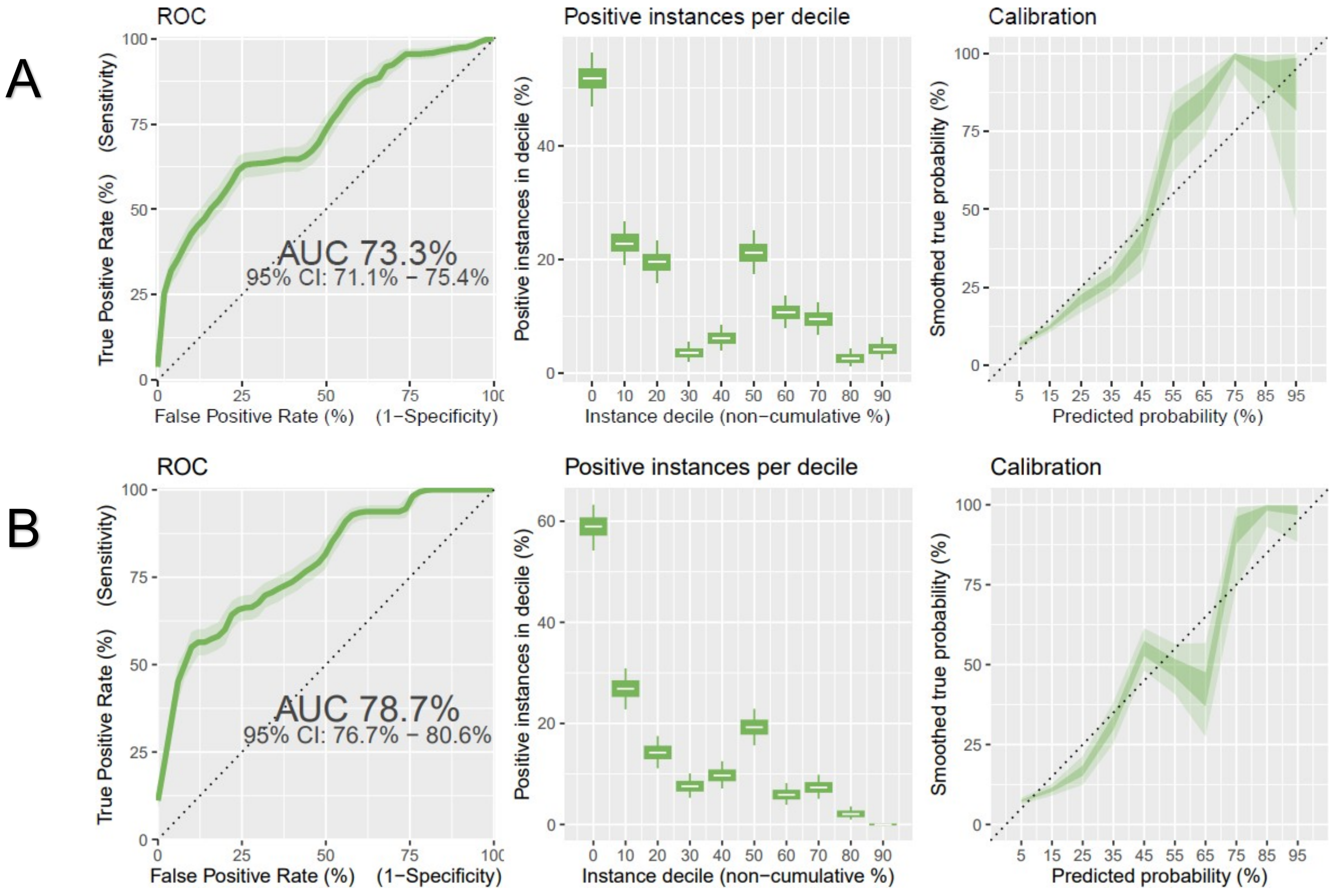

Figure S2. Model calibration. Calibration plots of the preoperative (**A**) and postoperative model (**B**). ROC curves as well as calibration plots with probabilities cut to deciles and as continuous variables are shown.
